# Rapamycin Ameliorates Inflammation and Fibrosis in the Early Phase of Cirrhotic Portal Hypertension in Rats through Inhibition of mTORC1 but Not mTORC2

**DOI:** 10.1371/journal.pone.0083908

**Published:** 2014-01-03

**Authors:** Weijie Wang, Jiqi Yan, Huakai Wang, Minmin Shi, Mingjun Zhang, Weiping Yang, Chenghong Peng, Hongwei Li

**Affiliations:** 1 Department of Surgery, Ruijin Hospital, Shanghai Jiaotong University School of Medicine, Shanghai, China; 2 Key Laboratory of Systems Biomedicine verified by Ministry of Education, Shanghai Institute of Digestive Surgery, Ruijin Hospital, Shanghai Jiaotong University School of Medicine, Shanghai, China; University of Navarra School of Medicine and Center for Applied Medical Research (CIMA), Spain

## Abstract

**Objective:**

Hepatic stellate cells (HSCs) transdifferentiation and subsequent inflammation are important pathological processes involved in the formation of cirrhotic portal hypertension. This study characterizes the pathogenetic mechanisms leading to cholestatic liver fibrosis and portal hypertension, and focuses on mammalian target of rapamycin (mTOR) pathway as a potential modulator in the early phase of cirrhotic portal hypertension.

**Methods:**

Early cirrhotic portal hypertension was induced by bile duct ligation (BDL) for three weeks. One week after operation, sham-operated (SHAM) and BDL rats received rapamycin (2 mg/kg/day) by intraperitoneal injection for fourteen days. Vehicle-treated SHAM and BDL rats served as controls. Fibrosis, inflammation, and portal pressure were evaluated by histology, morphometry, and hemodynamics. Expressions of pro-fibrogenic and pro-inflammatory genes in liver were measured by RT-PCR; alpha smooth muscle actin (α-SMA) and antigen Ki67 were detected by immunohistochemistry; expressions of AKT/mTOR signaling molecules, extracellular-signal-regulated kinase 1/2 (ERK1/2), p-ERK1/2, and interleukin-1 beta (IL-1β) were assessed by western blot.

**Results:**

The AKT/mTOR signaling pathway was markedly activated in the early phase of cirrhotic portal hypertension induced by BDL in rats. mTOR blockade by rapamycin profoundly improved liver function by limiting inflammation, fibrosis and portal pressure. Rapamycin significantly inhibited the expressions of phosphorylated 70KD ribosomal protein S6 kinase (p-P70S6K) and phosphorylated ribosomal protein S6 (p-S6) but not p-AKT Ser473 relative to their total proteins in BDL-Ra rats. Those results suggested that mTOR Complex 1 (mTORC1) rather than mTORC2 was inhibited by rapamycin. Interestingly, we also found that the level of p-ERK1/2 to ERK1/2 was significantly increased in BDL rats, which was little affected by rapamycin.

**Conclusions:**

The AKT/mTOR signaling pathway played an important role in the early phase of cirrhotic portal hypertension in rats, which could be a potential target for therapeutic intervention in the early phase of such pathophysiological progress.

## Introduction

Liver cirrhosis is a very complex disease in which multiple pathological processes are closely involved, including inflammatory infiltration and fibrogenesis [1], [2]. In fact, liver fibrosis represents a wound-healing process in response to a variety of chronic stimuli, which is characterized by an excessive deposition of extracellular matrix proteins (ECM) [3], [4]. Induction of secondary biliary cirrhosis by bile duct ligation is a widely used model to investigate the pathophysiological changes that take place during the development of hepatic fibrogenesis and portal hypertension [5]–[8]. Along with cholangiocytes proliferation, quiescent HSCs transform into proliferative, fibrogenic, and contractile myofibroblasts, and then produce bulk of ECM, predominated by type I collagen [6], [8]. They are both key players in the development of cholestatic liver fibrosis and portal hypertension [5], [8].

To date, there is still limited specific medical therapy for hepatic fibrosis and portal hypertension. The precise pathogenetic mechanisms of cirrhotic portal hypertension have not yet been made entirely clear. Mammalian target of rapamycin (mTOR) and AKT, also called protein kinase B (PKB), are serine/threonine protein kinases akin to the phosphatidylinositol 3-kinase-related kinase (PI3K) protein family [9]–[11]. One of the functions of AKT is activation and phosphorylation of mTOR [9], [11], [12]. AKT/mTOR signaling pathway, the major downstream effector of PI3K, regulates a wide array of cellular processes such as cell growth, proliferation, motility, survival, apoptosis, protein synthesis and transcription [9], [12]–[14]. Emerging experimental data showed that AKT/mTOR signaling pathway positioned itself at the center in the activation of hepatic stellate cells (HSCs) [3], [15], [16], and the pathway blockade by rapamycin could reduce fibrogenesis, improve liver function, and lower portal pressure in established cirrhotic animal models [5]–[7]. Therefore, we became especially interested in its conceivable mechanisms during portal hypertension’s early pathophysiologic progress.

mTOR is the catalytic subunit of two molecular complexes: mTOR Complex 1 (mTORC1) and Complex 2 (mTORC2). They have distinct substrate specificities and are differentially sensitive to rapamycin, therefore differentially regulated [14], [17]. mTORC1 integrates signals from growth factor receptors, then activates the 40S ribosomal protein S6 kinase (P70S6K) and inhibits the eukaryotic initiation factor (eIF) 4E-binding protein-1 (4E-BP1) by phosphorylation, forming two parallel signaling pathways regulating mRNA translation to control protein synthesis [14], [18]. mTORC2 appears to possess the activity to phosphorylate the serine/threonine protein kinase AKT/PKB at a serine residue Ser473 [2]. Phosphorylation of the serine residue stimulates AKT phosphorylation at a threonine Thr308 residue by phosphoinositide-dependent kinase-1 (PDK1) and leads to full AKT activation [2], [17].

Rapamycin as a bacterial macrolide with antifungal and immunosuppressive properties forms a complex with the FK binding protein (FKBP-12) that binds with high affinity to mTOR [19]. Several documents showed that rapamycin could inhibit the activation and proliferation of HSCs in vitro [15], [20], [21]. Mejias and Fernandez also confirmed that rapamycin had the inhibitory effect on lymphocyte proliferation, neovascularization and fibrogenesis in splenomegaly, as well as on the development of pathological angiogenesis in mesenteric tissues [22], [23]. However, there is still little known about the specific role of the AKT/mTOR signaling pathway in the early pathophysiologic progress of cirrhotic portal hypertension. Comprehensive and in-depth researches on the pathway in cirrhotic portal hypertension have rarely been reported. We hypothesized that AKT/mTOR signaling pathway was activated in the pathophysiological onset of early cirrhotic portal hypertension, and rapamycin treatment would have multiple mechanisms of action against hepatic fibrosis and portal hypertension progression. Thereafter, we explored its potential therapeutic mechanisms in an animal model of early cirrhotic portal hypertension.

## Materials and Methods

### Ethics Statement

All animal care and experimental procedures complied with the guidelines for the Care and Use of Laboratory Animals, formulated by the Ministry of Science and Technology of the People’s Republic of China, were approved by the Ethical Committee on Animal Experiments at Ruijin Hospital (protocol approval number SYXK 2011-0113).

### Materials

Antibody against α-smooth muscle actin (α-SMA) was from Doka (Denmark). Antibody against interleukin 1 beta (IL1-β) and Ki67 were bought from Santa Cruz Biotechnology (USA). Antibody against p-ERK1/2 was purchased from R&D (USA). Antibodies against AKT, mTOR, P70S6K, ribosomal protein S6 and their phosphorylated forms, including total protein ERK1/2, as well as peroxidase-conjugated secondary antibodies, were from Cell Signaling Technology (USA). Antibody against β-Actin was from Sigma (USA). Rapamycin was kindly donated by Pfizer Inc (USA).

### Animals and Rapamycin Treatment Schedule

Liver cirrhotic portal hypertension was induced in male Sprague-Dawley rats (230–250 g body weight) by bile duct ligation (BDL). Sham-operated (SHAM) rats underwent the same surgical procedure, with the bile duct being isolated and manipulated, but not ligated. Rapamycin (5% DMSO solution with rapamycin; 2 mgkg^−1^ day^−1^; BDL-Ra: n = 12; SHAM-Ra: n = 10) or vehicle (5% DMSO solution; BDL: n = 12; SHAM: n = 10) was administered intraperitoneally for a 2-week period, starting one week after ligation, when it was still at an early stage of cirrhotic portal hypertension.

### Haemodynamic Measurements, Liver Function and Tissue Conservation

At the end of the respective treatment phases, haemodynamic measurements - heart rate (HR) and mean arterial blood pressure (MBP) - were monitored by Tail-cuff method (BP-98A, Softron, Japan) in awake rats, and portal vein pressure (PP) was performed under pentobarbital anaesthesia. To maintain stable anesthesia, the measurement was started 20 min after the injection of pentobarbital. A segment of the mesenteric branch vein was cannulated with a 24-g cannula needle, and the tip of the cannula was advanced just into the trunk of the superior mesenteric vein. The PP was measured via a pressure transducer and recorded by a multichannel computer-based recorder (PowerLab, ADInstruments, Australia). Pressure measurement lasted for 1 min, and the average value was regarded as the portal pressure (PP). Rats were killed by exsanguination while taking blood samples for analyzing the serum levels of aspartate aminotransferase (AST), alanine aminotransferase (ALT), alkaline phosphatase (ALP), total bilirubin (TB), bile acids (BA), creatinine (CREA) and albumin (ALB). All of which were measured in the Department of Clinical Chemistry of Ruijin Hospital, Shanghai Jiaotong University School of Medicine. Liver and spleen were removed and weighed. Liver tissue samples were either fixed in 10% formalin, 4% glutaraldehyde or stored at −80°C.

### Histology and Immunohistochemistry

Glutaraldehyde-fixed liver tissue was used for transmission electron microscope examination to observe the microscopic changes in the intrahepatic cells. Formalin-fixed and paraffin-embedded liver tissue was used for evaluation of liver pathological changes after HE, Masson and immunohistochemistry staining.

For immunohistochemistry, antigen retrieval was performed with citrate buffer (pH 6.0; 15 min at 95°C), followed by blocking with 0.6% H_2_O_2_ and 2% goat serum. Antibodies against α-SMA (1∶400) and Ki67 (1∶200) were applied 37°C for 1–2 hours, followed by biotinylated the corresponding secondary antibodies (DAKO, Denmark) at 1∶350 for 45 min at room temperature (RT), streptavidin-HRP for 30 min at RT, and diaminobenzidine (DAB) detection. HE-, Masson- and DAB-stained liver tissue slides were analysed by morphometry. Morphometry was performed from three random fields of every slide using the Image-Pro plus software (Media Cybernetics Inc, USA). For each microscopic field, the positive area was calculated automatically by the software, and this positive area was in turn divided by the total area of the microscopic field. Morphometric results were then expressed as volume fractions (percentage of specific positive area in relation to the total counted area) and given as means ±SEM.

### RNA Isolation and Quantitative Real-time PCR

Total RNA was isolated from about 30 mg of liver tissue with Trizol Reagent (Invitrogen, USA) according to the manufacturer’s instructions. Then reverse transcription was done using Reverse Transcription Kit (Promega, USA). Quantitative real-time PCR was carried out on a 7900HT Fast Real-Time PCR System (Applied Biosystems, USA). The rat nucleotide sequences and accession numbers of primers were summarized in Table 1. Data were normalized to the housekeeping gene glycerinaldehyd-3-phosphatdehydrogenase (GAPDH).

**Table 1:** Nucleotide sequences of RT-PCR primers.

Target mRNA	Direction Sequence (Forward/Reverse)	Accession Number
**GAPDH**	GAGGACCAGGTTGTCTCCTG	NM 017008.4
	GGATGGAATTGTGAGGGAGA	
**mTOR**	GACAACAGCCAGGGCCGCAT	NM 019906.1
	ACGCTGCCTTTCTCGACGGC	
**P70S6K**	CGGGGAAGCTTCAGCGCCAC	NM 031985.1
	TACGCAGGTGCTCTGGCCGT	
**4EBP1**	CTGCACAGCAGCCCGGAAGA	NM 053857.2
	TGCCGGGTACAAGGCCTGACT	
**PCα1**	TCCGGCTCCTGCTCCTCTTA	NM 053304.1
	GTATGCAGCTGACTTCAGGGATGT	
**α-SMA**	GCTGACAGGATGCAGAAGGA	NM 031004.2
	GCCGATCCAGACAGAATATTTG	
**PDGF**	CCGCTCCTTTGATGACCTTC	NM 031524.1
	GCTCAGCCCCATCTTCGTC	
**PDGFRβ**	TGGCCAATGGCATGGAAT	NM 031525.1
	CCAACTTGCCCTCACAGATGA	
**TGF β1**	AGAAGTCACCCGCGTGCTAA	NM 021578.2
	TCCCGAATGTCTGACGTATTGA	
**TIMP 1**	TCCTCTTGTTGCTATCATTGATAGCTT	NM 053819.1
	CGCTGGTATAAGGTGGTCTCGAT	
**TNF-α**	GACCAGGCTGTCGCTACATCA	NM 012675.3
	GTAGGGCGATTACAGTCACGG	
**iNOS**	AGCGGCTCCATGACTCTCA	NM 012611.3
	AAATGCTTTCTCCGCTCTGA	

The results were expressed as the number of cycles (CT value) at which the fluorescence signal exceeded a defined threshold. The difference in CT values of the target cDNA and GAPDH was expressed as ΔCT values. Therefore, lower ΔCT values denoted higher mRNA levels. The 2^−ΔCT^ method was used for quantification of the results.

### Western Blot Analysis

Tissue samples were put in ice-cold Tissue Protein Extraction Reagent (Pierce, USA) containing a mixture of proteinase inhibitors cocktails (Sigma, USA) and phosphatase inhibitors (Roche, Switzerland) to be homogenated by the tissue homogenizer. Samples were then centrifuged for 30 minutes at 10,000 g. The supernatant was collected, and the protein concentration was measured using the BCA Protein Assay Kit (Pierce, USA). Proteins (80∼120 µg) were separated by SDS–PAGE and subsequently transferred to polyvinylidenefluoride membranes. The membranes were blocked with 5% bovine serum albumin (BSA) in incubation buffer for 2 h at room temperature. Afterwards, they were incubated with the corresponding primary antibodies at 4°C overnight and then with the peroxidase-conjugated secondary antibody at room temperature for 2 h. Finally, the signal was detected by enhanced chemiluminescence. Loading accuracy was evaluated by membrane rehybridization with anti-β-Actin antibodies. ImageJ software was used to quantify western blot signals.

### Statistics

Comparison between multiple groups was performed with the one-way ANOVA Kruskal–Wallis test, and pairwise comparison with Mann Whitney U Test using the Graph Pad statistics software (Graph Pad Software Inc, USA). Results were considered statistically significant with a p-value <0.05. Results in the Tables were given as means ± standard deviation (SD). The columnar diagram results indicated means ± standard error of the mean (SEM).

## Results

### Rapamycin Ameliorates Liver Size, Splenomegaly and Portal Pressure

**Figure 1:**
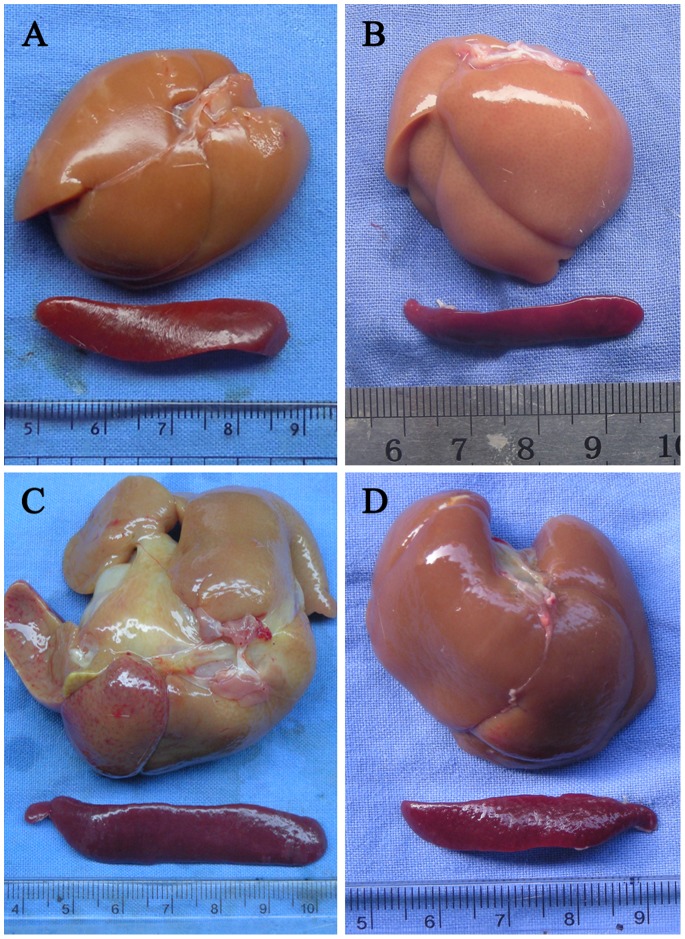
Rapamycin treatment ameliorates liver size and splenomegaly in BDL-Ra rats. Representative photographs of liver and spleen from (A) SHAM, (B) SHAM-Ra, (C) BDL, (D) BDL-Ra rats.

BDL for three weeks markedly impaired body weight gain by 15.2% (p<0.005) and significantly resulted in increased liver (p<0.005) and spleen (p<0.005) size compared with SHAM rats (Figure 1). The ratio of spleen weight to body weight, which is a measure of organ size, markedly increased by 1.6-fold (p<0.005) in BDL compared with SHAM rats, indicating the formation of portal hypertension. However, treatment with rapamycin for fourteen days obviously altered not only the growth of the body, but also the organ weights in the BDL-Ra animals (Table 2). Compared with SHAM and BDL animals, SHAM-Ra and BDL-Ra groups reduced body weight gain by 22.5% (p<0.005) and 14.7% (p<0.005) respectively. No significant differences were obtained comparing SHAM rats with SHAM-Ra animals in relative liver weight. However, liver size of BDL rats was clearly higher than that of SHAM (p<0.005) and BDL-Ra (p<0.005) groups. Rapamycin also profoundly ameliorated splenomegaly in BDL-Ra rats (p<0.005). These results demonstrated that rapamycin treatment attenuated the increase in liver and spleen size that typically succeeded BDL.

**Table 2:** Animal characteristic and hemodynamic parameters.

Indicators	SHAM (n = 10)	SHAM-Ra (n = 10)	BDL (n = 12)	BDL-Ra (n = 12)	P
**BW (g)**	340.90±24.00***	263.60±15.47###	289.30±35.56	247.70±25.93***	<0.0001
**LW (LW/BW%)**	4.05±0.46***	4.18±0.34	8.33±1.45	5.73±0.69***	<0.0001
**SW (SW/BW%)**	0.26±0.06***	0.18±0.02###	0.68±0.16	0.30±0.06***	<0.0001
**HR (bpm)**	376.20±26.24**	438.50±41.3	393.50±29.19	405.00±26.24**	<0.0001
**MBP (mmHg)**	104.30±10.96***	130.30±10.17	117.30±13.49	119.70±12.24	<0.0001
**PP (mmHg)**	8.71±0.93***	9.07±1.39	15.78±1.97	11.58±2.10***	<0.0001

**Abbreviations:** BW, body weight; LW, relative liver weight; SW, relative spleen weight; HR, heart rate; MBP, mean arterial blood pressure; PP, portal pressure.

p<0.05;

p<0.01;

p<0.005 vs. BDL;

^###^ P<0.005 vs. SHAM (mean±SD; Pairwise comparison with Mann Whitney U Test; P, non-parametrical one-way ANOVA, Kruskal-Wallis Test).

As the Table 2 showed the hemodynamic parameters were favorably influenced by the rapamycin treatment. MBP was higher in the BDL (P<0.005) than that in SHAM animals but not significantly different between BDL and BDL-Ra groups, which was highest in the SHAM-Ra group. Consistent with splenomegaly, portal pressure was markedly elevated in BDL rats compared with SHAM controls (P<0.005). However, it was decreased by 26.6% (P<0.005) with rapamycin treatment, with no significant difference between SHAM and SHAM-Ra rats. Detailed values were given in Table 2.

### Rapamycin Reduces Cholangiocytes Proliferation and ECM Deposition, as Well as Improves Liver Function

**Figure 2:**
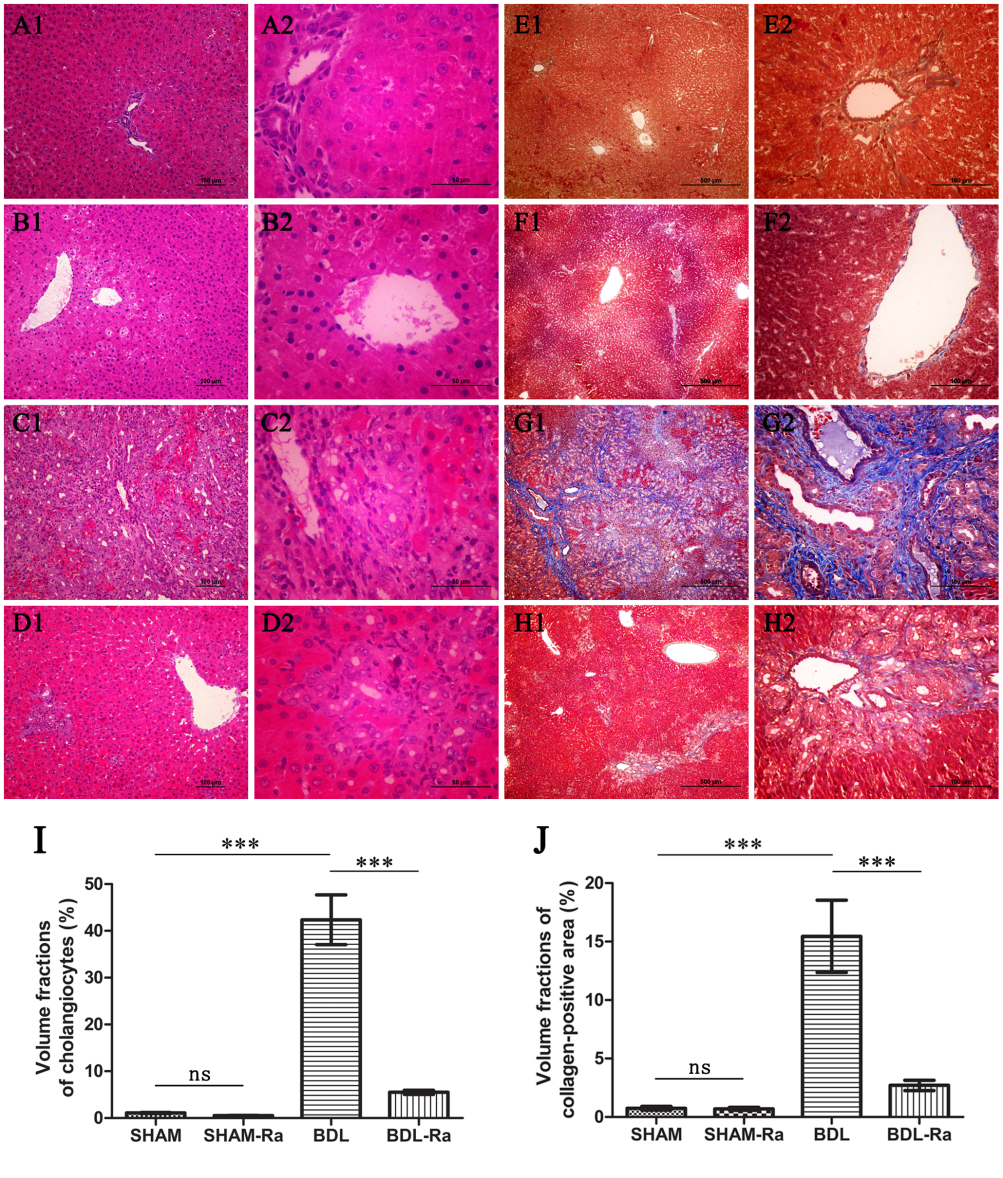
Rapamycin treatment reduces cholangiocytes proliferation and ECM deposition in BDL-Ra rats. Representative liver sections of (A) SHAM, (B) SHAM-Ra, (C) BDL, and (D) BDL-Ra rats, following HE-staining (magnification 100× and 400×, respectively). Representative microphotographs of Masson-stained liver sections from (E) SHAM, (F) SHAM-Ra, (G) BDL, and (H) BDL-Ra rats (magnification 40× and 200×, respectively). Graphs (I) and (J): Quantitative analysis of HE and Masson staining in representative liver sections respectively (mean±SEM; *p<0.05; **p<0.01; ***p<0.005; ns - nonsignificant).

The histological detection demonstrated that rapamycin treatment did not affect morphometry in SHAM and SHAM-Ra rats, but significantly attenuated cholangiocytes proliferation as well as the ECM deposition in BDL-Ra rats (Figure 2). Statistics of HE and Masson staining under the same conditions showed that volume fractions of bile ducts and ECM correspondingly decreased by 87.0% (P<0.005) and 82.5% (P<0.005) in BDL-Ra group compared with BDL rats.

Paralleled by an inhibition of the histological changes succeeding BDL, rapamycin also improved liver function greatly (Table 3). We could easily observe a poor liver function in BDL rats. However, treatment with rapamycin distinctly decreased ALT (P<0.005), AST (P<0.005), ALP (P<0.01), TB (P<0.05), and BA (P<0.01) in BDL-Ra group compared with BDL rats, but had little influence on ALB, and CREA, indicating the therapeutic effect of rapamycin on liver function. The data also showed no significant differences in ALP, AST, TB, ALB, and CREA between SHAM and SHAM-Ra animals. But the ALP of SHAM-Ra was higher (P<0.05) and the BA was lower (P<0.05) than those in SHAM.

**Table 3:** Serum enzymes.

	SHAM (n = 8)	SHAM-Ra (n = 8)	BDL (n = 12)	BDL-Ra (n = 12)	P
**ALT**	52.58±6.25***	57.80±19.28	200.40±123.70	78.00±13.73***	<0.0001
**AST**	160.80±23.19***	191.40±68.38	759.50±271.10	323.80±163.10***	<0.0001
**ALP**	324.60±65.49*	245.20±63.86^#^	420.60±79.80	316.00±86.17**	0.0011
**TB**	5.75±1.18***	9.12±3.59	158.00±27.64	122.90±49.39*	<0.0001
**BA**	13.46±7.46***	23.26±9.03^#^	109.70±14.71	73.37±33.79**	0.0007
**CREA**	17.22±3.12**	22.27±5.67	46.44±29.22	27.75±18.43	<0.0001
**ALB**	14.79±0.98**	11.20±2.14	10.13±1.99	9.00±1.91	0.0322

**Abbreviations:** ALT, alanine aminotransferase (IU/L); ALP, alkaline phosphatase (IU/L); AST, aspartate aminotransferase (IU/L); BA, bile acids (µmol/L); TB, total bilirubin (µmol/L); CREA, creatinine (µmol/L); ALB, albumin (g/L). (mean±SD; *p<0.05; **p<0.01; ***p<0.005 vs. BDL; ^#^P<0.05 vs. SHAM; Pairwise comparison with Mann Whitney U Test; P, non-parametrical one-way ANOVA, Kruskal-Wallis Test).

### Rapamycin Reveals Inverse Effects on Fibrosis- and Inflammatory-related mRNA

Multiple pathological processes such as inflammatory infiltration and fibrogenesis are closely involved in the development of hepatic fibrosis. Since AKT/mTOR signaling pathway plays a central role in immunological processes and fibrogenesis [22], we next assessed mTOR signaling molecules, intrahepatic inflammation and fibrosis at transcription level.

**Figure 3:**
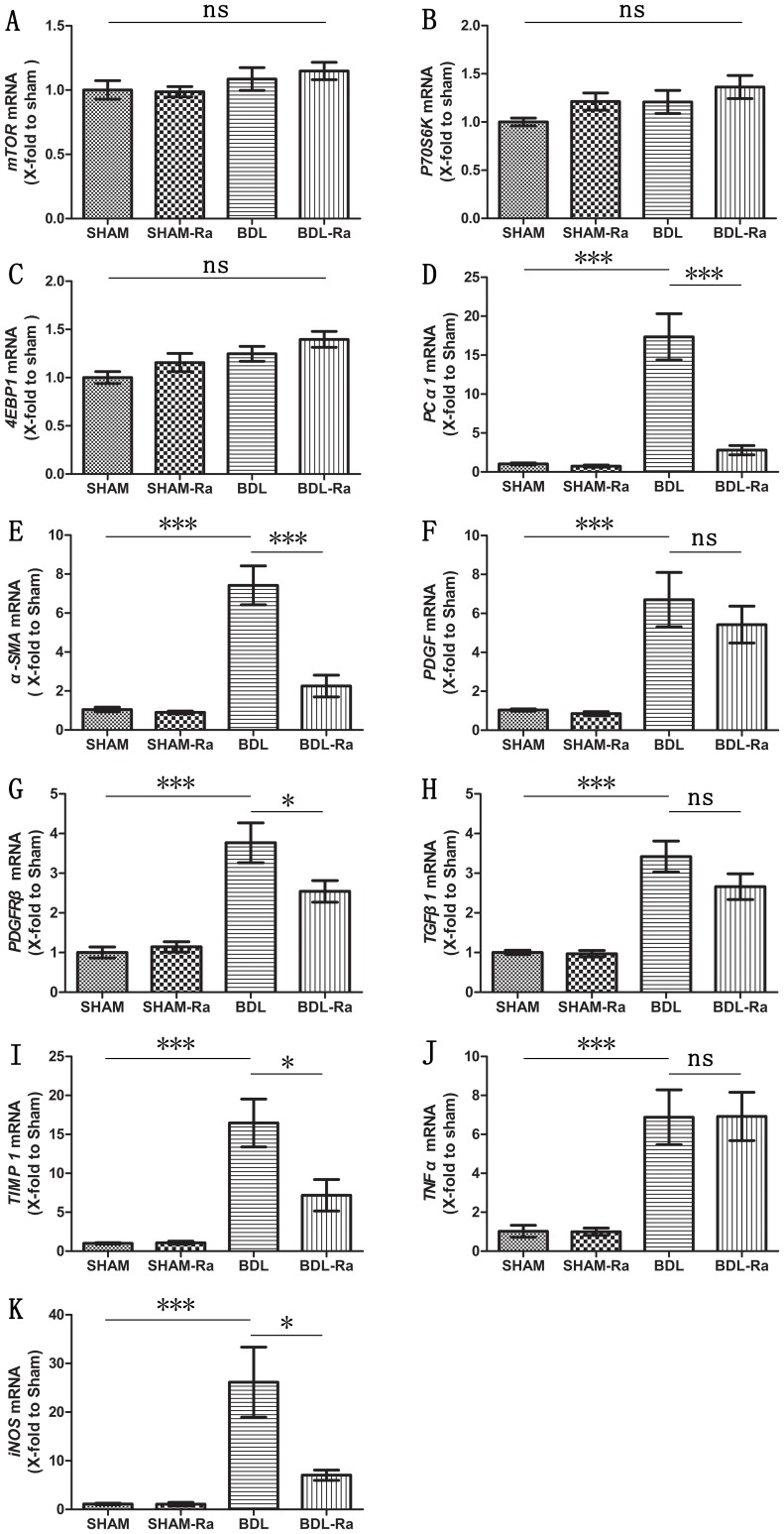
Rapamycin treatment downregulates liver fibrosis- and inflammatory-related genes in BDL-Ra rats. (A) mTOR, (B) P70S6K, (C) 4EBP1, (D) Procollagen (PC) α1(I), (E) α-SMA, (F) PDGF, (G) PDGFRβ, (H) TGF β1, (I) TIMP-1, (J) TNF-α, and (K) iNOS mRNA, measured by fluorescence quantitative PCR and normalized to GAPDH (mean±SEM; *p<0.05; **p<0.01; ***p<0.005; ns - nonsignificant).

RT-PCR results showed that mRNA expressions of the mTOR, P70S6K and 4EBP1 were not significantly different among the four groups and not obviously affected by rapamycin. Our findings however showed that profibrogenic genes such as α-SMA, procollagen α1(I) (PC α1), transforming growth factor beta 1 (TGF β1), platelet-derived growth factor (PDGF), PDGF-receptor beta (PDGFRβ), and tissue inhibitor of metalloproteinase 1 (TIMP-1), as well as the proinflammatory genes tumor necrosis factor alpha (TNF-α) and inducible nitric oxide synthase (iNOS) were significantly upregulated approximately 6.1-, 16.0-, 2.4-, 5.5-, 2.8-, 15.5-, 5.7- and 22.2-fold in the BDL rats respectively compared with the SHAM group (Figure 3). Treatment with rapamycin reduced mRNA expressions of the α-SMA, PC α1, PDGFRβ, TIMP-1, and iNOS by 69.6% (P<0.005), 83.9% (P<0.005), 32.5% (P<0.05), 56.3% (P<0.05) and 73.1% (P<0.05) in the BDL-Ra rats. While genes of PDGF, TGF β1, and TNF-α were not significantly different between BDL and BDL-Ra groups, rapamycin showed a beneficial tendency for downregulating the genes of PDGF and TGF β1 with the exception of TNF-α. In addition, no striking difference was acquired between SHAM and SHAM-Ra groups. Primer sequences information detailed in Table 1.

### Rapamycin Improves Liver Fibrosis and Inflammation

Along with the biliary ducts hyperplasia, the transdifferentiation and proliferation of HSCs mainly contributed to the development of hepatic fibrosis in cholestatic liver diseases.

**Figure 4:**
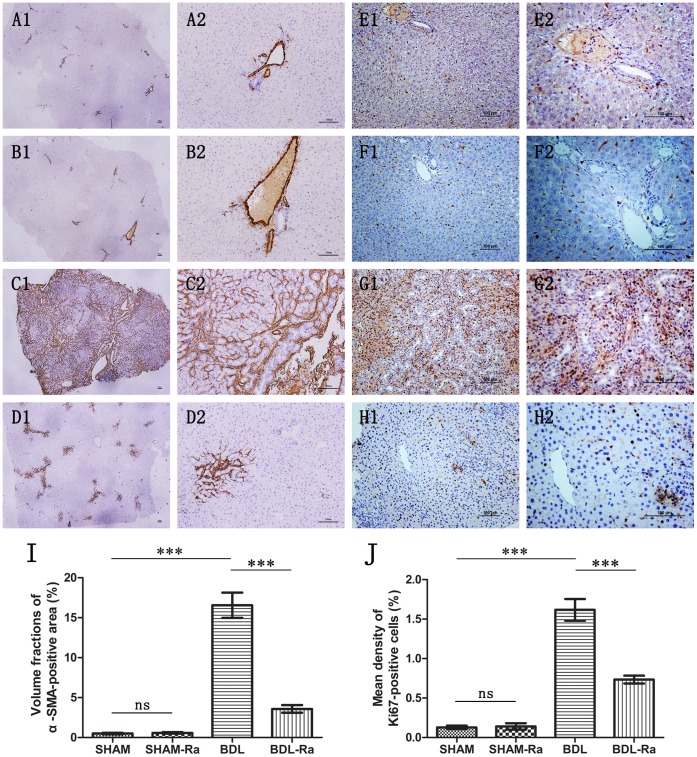
Rapamycin treatment decreases α-SMA and Ki67 expression in livers of BDL-Ra rats. Representative liver microphotographs of (A) SHAM, (B) SHAM-Ra, (C) BDL, and (D) BDL-Ra, following immunohistochemistry staining of α-SMA (magnification 40× and 100×, respectively). Representative liver sections of (E) SHAM, (F) SHAM-Ra, (G) BDL, and (H) BDL-Ra, using anti-ki67 for immunohistochemistry (magnification 100× and 200×, respectively). Graphs (I) and (J): Quantitative analysis of α-SMA- and Ki67-positive cells in representative liver sections respectively (mean±SEM; *p<0.05; **p<0.01; ***p<0.005; ns - nonsignificant).

Our results showed the expression of Ki67, a nuclear protein expressed only in proliferating cells, was makedly increased in BDL rat livers. Treatment with rapamycin evidently reduced the Ki67-positive cells in BDL-Ra rats, indicating the suppression of intrahepatic cell proliferation by rapamycin (Figure 4). α-SMA is commonly used as a marker of activated HSCs [3], [6]–[8]. We next found that α-SMA-positive cells appeared around the proliferative bile ducts, which increased significantly in livers of BDL compared with those of SHAM groups. More importantly, bile ducts proliferation as well as numbers of periductular α-SMA-positive cells were significantly reduced by rapamycin (Figure 4), which was consistent with our Masson staining.

**Figure 5:**
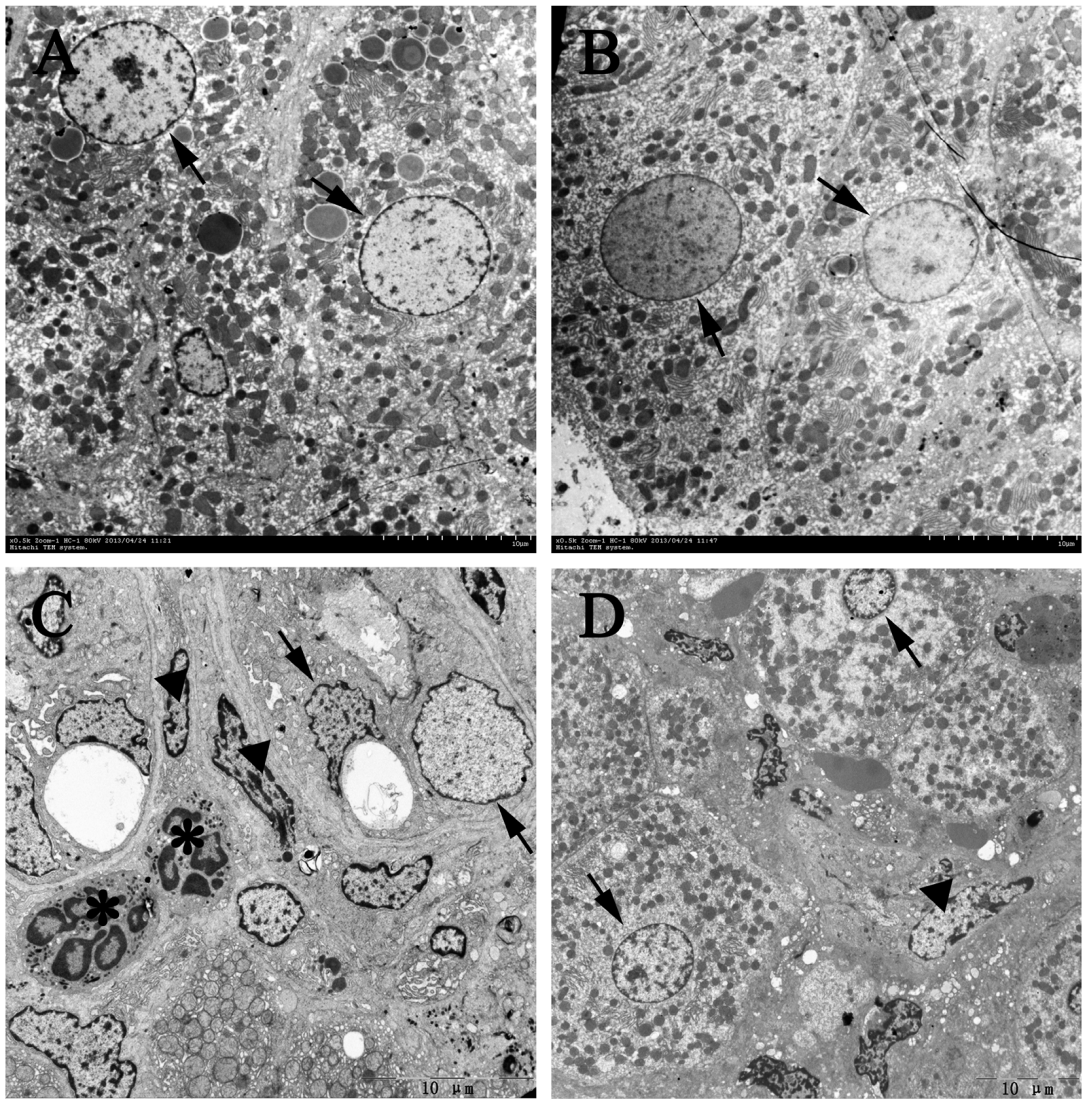
Rapamycin treatment improves the intrahepatic microstructure in BDL-Ra rats, evaluated by transmission electron microscopy (TEM). Representative liver TEM micrographs of (A) SHAM, (B) SHAM-Ra, (C) BDL, (D) BDL-Ra (magnification A, B: 5000×; C, D: 3000×, respectively). Arrow heads point to liver nuclei, triangles represent fibroblasts and asterisks denote neutrophils.

Moreover, enhanced inflammation plays an important role in early stage of cholestatic liver injury and always aggravates the fibrogenesis processes [1], [24]. Our electron microscopy results showed no significant differences between SHAM and SHAM-Ra. The hepatocytes had intact structure, and seemed nearly normal in cytoplasm and interstitial. In contrast, the shape of the hepatocyte nuclei became irregular, and some even appeared pyknotic in the BDL group. The mitochondria partly disappeared in damaged hepatic cytoplasm, with vacuolar degeneration visible occasionally. Furthermore, the structure of hepatic interstitial was disordered, where bile duct endothelial cells, fibroblasts, macrophages, lymphocytes and neutrophils obviously increased. The normal structure of sinusoidal endothelial fenestrae also disappeared. However, treatment with rapamycin reduced the amount of intrahepatic neutrophils, lymphocytes, fibroblasts and ECM, as well as hepatocellular injury (Figure 5).

**Figure 6:**
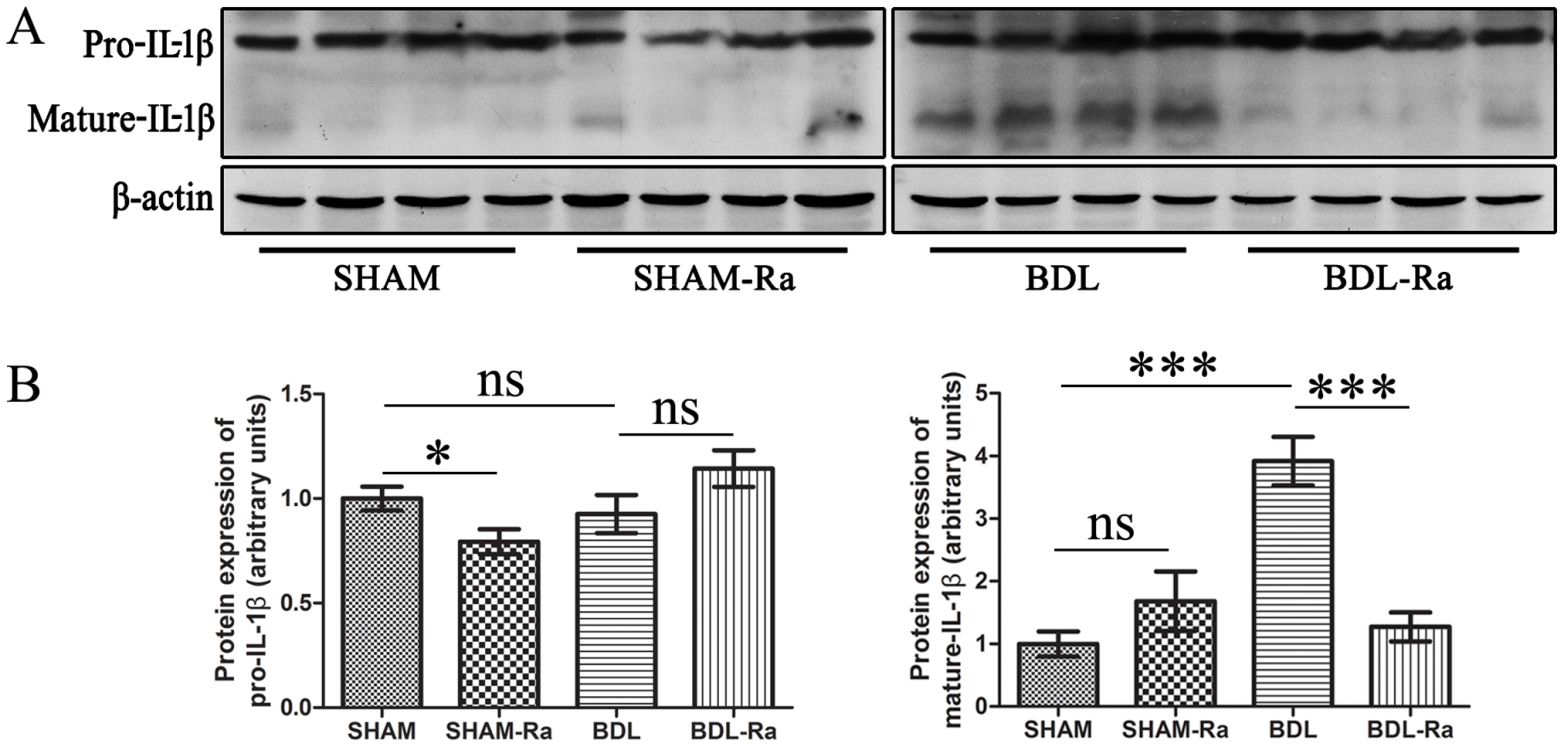
Rapamycin treatment attenuates intrahepatic inflammation in BDL-Ra rats. Representative western blot (A) and densitometric analyses (B) for precursor- and mature-IL-1β expressions in rat livers respectively (mean±SEM; *p<0.05; **p<0.01; ***p<0.005; ns - nonsignificant).

IL-1β is one of proinflammatory cytokines thought to be involved in many acute and chronic diseases [25], [26]. It is produced by a variety of cells, including macrophages, monocytes, and keratinocytes [27]. IL-1β activity is tightly controlled and requires the conversion of the primary transcript, the inactive IL-1β precursor, to the active mature IL-1β by limited proteolysis. Our western blot result suggested that while the IL-1β precursor expression was not significantly different between BDL and BDL-Ra groups, the mature IL-1β evidently increased in BDL livers, indicating inflammation was involved in the pathophysiological onset and being consistent with the neutrophil infiltration showed by electron microscopy. Rapamycin might suppress the tranformation from IL-1β precursor to mature IL-1β and then markedly decreased mature IL-1β (Figure 6).

Taken together, these results indicated that the molecular basis for the rapamycin induced improvement in BDL-Ra rats involved reduction of HSCs and cholangiocytes proliferation, as well as the blockade of inflammatory process at least in part.

### The Role of AKT/mTOR Signaling Pathway in the Early Phase of Cirrhotic Portal Hypertension

**Figure 7:**
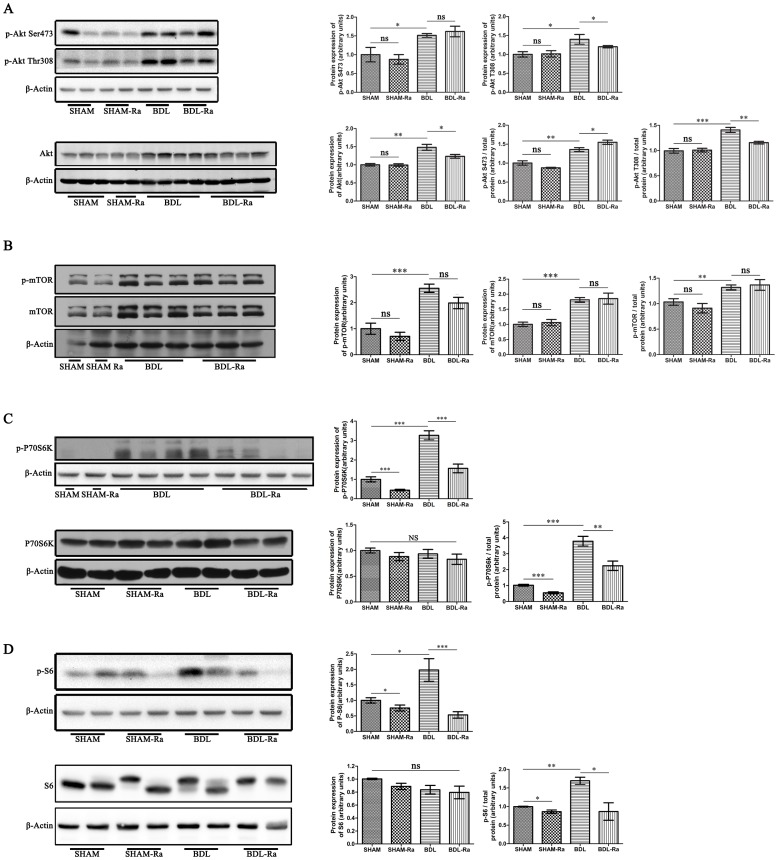
Rapamycin treatment blocks mTORC1 but not mTORC2 in rat livers. Representative immunoblot and densitometric analyses of hepatic (A) p-AKT and AKT, (B) p-mTOR and mTOR, (C) p-P70S6K and P70S6K, and (D) p-S6 and S6 in rat livers respectively (mean±SEM; *p<0.05; **p<0.01; ***p<0.005; ns - nonsignificant).

AKT is an important upstream mediator of mTOR and also regulated by mTORC2 [2]. We detected, using the western blot technique, a significant upregulation of the p-AKT Ser473 and Thr308 to total protein in the livers of BDL rats compared with SHAM animals (Figure 7A). The levels of the immediate downstream target proteins p-mTOR, p-P70S6K and p-S6 to their total proteins were also higher in the livers from BDL rats than those from SHAM animals (Figure 7BCD). Moreover, our results suggested that the expressions of AKT and mTOR were also increased, with the exception of P70S6K and S6. Taken these results together, we firstly demonstrated that AKT/mTOR signaling pathway was significantly overactivated and might be involved in the early pathogenesis of cirrhotic portal hypertensive rats.

To gauge the impact of targeting mTOR pathway on liver fibrosis and portal hypertension induced by BDL, we treated BDL rats with the mTOR inhibitor rapamycin for two weeks. Chronic rapamycin treatment efficiently downregulated the mTOR signaling pathway in the livers of BDL-Ra rats, as showed by the downward trend of p-mTOR and the significant reduction of p-P70S6K and p-S6 to their total proteins compared with BDL rats (Figure 7BCD). Similarly, the mTOR signaling pathway in SHAM-Ra rats was also evidently inhibited by rapamycin compared with SHAM group (Figure 7CD). In addition, we found no significant differences in the relative expressions of p-AKT (Ser473) and p-AKT (Thr308) between SHAM and SHAM-Ra group. However, the relative expression of p-AKT Thr308 in BDL-Ra rats was decreased compared with BDL group (Figure 7A). In contrast, the expression of p-AKT (Ser473) to AKT in BDL-Ra was much higher than that in BDL groups. Besides, rapamycin had little effects on the expressions of mTOR, P70S6K, and S6 compared with SHAM and BDL groups respectively, as well as on the expression of AKT between SHAM and SHAM-Ra groups. While rapamycin significantly reduced the expression of AKT compared with BDL group, the level of AKT in BDL-Ra rats was still much higher than that in SHAM group.

### Rapamycin has no Evident Effect on the Level of p-ERK1/2 to ERK1/2

Many investigations suggested that there existed a cross talk between mitogen-activated protein kinase (MAPK)/extracellular signal-regulated kinase (ERK) and PI3K/mTOR pathways to co-regulate the proliferation and survival of the hepatocytes [20], [28]–[31], cholangiocytes [20], [32], [33] and HSCs [11], [15], [20], [34]. We also detected the expressions of p-ERK1/2 and ERK1/2 by western blot technique. We found that both the expressions of p-ERK1/2 and ERK1/2 increased in BDL rats, and rapamycin markedly decreased the expression of ERK1/2 in BDL-Ra rats, with the exception of p-ERK1/2.

**Figure 8:**
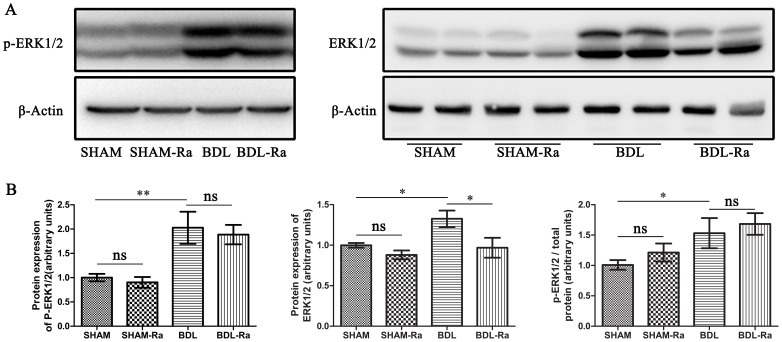
Rapamycin treatment has no evident effect on p-ERK1/2 expression in rat livers. Representative western blot (A) and densitometric analyses (B) of p-ERK1/2 and ERK1/2 in rat livers respectively (mean±SEM; *p<0.05; **p<0.01; ***p<0.005; ns - nonsignificant).

Moreover, the level of p-ERK1/2 to ERK1/2 in BDL rats was significantly higher than in SHAM animals (Figure 8), indicating that MAPK/ERK cascade was manifestly overactivated. However, treatment with rapamycin had no obvious effect on the expression of p-ERK1/2 to ERK1/2 compared with their controls respectively. Based on the therapeutic action of rapamycin, these data implied that AKT/mTOR singnaling did play a vital role in the formation of hepatic fibrosis and portal hypertension induced by BDL from another perspective.

## Discussion

Liver cirrhosis is the endpoint of the fibrogenic process in which inflammation and fibrogenesis are closely integrated [1]. Several studies have established a role for mTOR as an attractive target for portal hypertension and antifibrotic therapy [5]–[7], [19], [22], [35]. However, comprehensive and in-depth researches on the AKT/mTOR signaling pathway in cirrhotic portal hypertension have rarely been reported, especially ones on the early phase of this disease. In this study an early cirrhotic portal hypertensive rat model was successfully established at three weeks after BDL, when portal vein pressure and spleen size were significantly increased but intrahepatic nodules and fiber spacing had not yet formed (Figure 2). We extensively evaluated the early pathological processes and systematically explored AKT/mTOR signaling pathway in the early phase of cirrhotic portal hypertension.

The present study suggested that HSCs transdifferentiation, cholangiocytes proliferation and inflammatory infiltration mainly contributed to the development of hepatic fibrosis and portal hypertension in cholestatic liver fibrosis. HSCs play a crucial role in the process of hepatic fibrosis, as they are responsible for excessive deposition of ECM proteins [11], [15], amplification of inflammatory response by inducing infiltration of mono- and polymorphonuclear leukocytes [4], as well as increase in intrahepatic resistance [36], which is consistent with our Masson-staining result and the elevated portal pressure in BDL rats. Morphological change with HSCs activation is evident in appearance of the cytoskeletal protein α-SMA [3], [6]–[8]. Our RT-PCR and immunohistochemical detections consistently demonstrated a high expression of α-SMA in BDL rats, indicating the activation and proliferation of HSCs. In this regard, we even intuitively witnessed the fibroblasts transformed from HSCs and collagen fiber structure in the liver interstitial using electron microscopy. HE-staining and electron microscopy also showed a large number of proliferative cholangiocytes and infiltration of neutrophils in BDL rats.

PDGF and TGFβ1 are the most potent mitogen and stimulus for HSCs to produce ECM proteins respectively [5], [37]. The former is not only secreted in an autocrine fashion by HSCs but also synthesized by cholangiocytes during cholestasis. The latter is derived from both paracrine and autocrine sources of HSCs [5]. TIMP1, a tissue inhibitor of metalloproteinases, is a natural inhibitor of the matrix metalloproteinases (MMPs), a group of peptidases involved in degradation of the extracellular matrix. We demonstrated a significant up-regulation of the powerful profibrogenesis genes PDGF, PDGFRβ, TGF-β1, PC-α1 and TIMP1, as well as pro-inflammatory genes TNF-α and iNOS in the liver of cirrhotic portal hypertensive rats. Moreover, the western blot result of IL-1β further suggested an enhanced inflammation during the development of hepatic fibrosis. Treatment with rapamycin effectively ameliorated these pathological processes as well as improved liver function and portal pressure, implying that AKT/mTOR signaling pathway contributed to the early pathology of cirrhotic portal hypertension, consistent with the studies conducted by Reif on HSCs culture and by Neef on the established cirrhotic rat models [3], [6].

**Figure 9:**
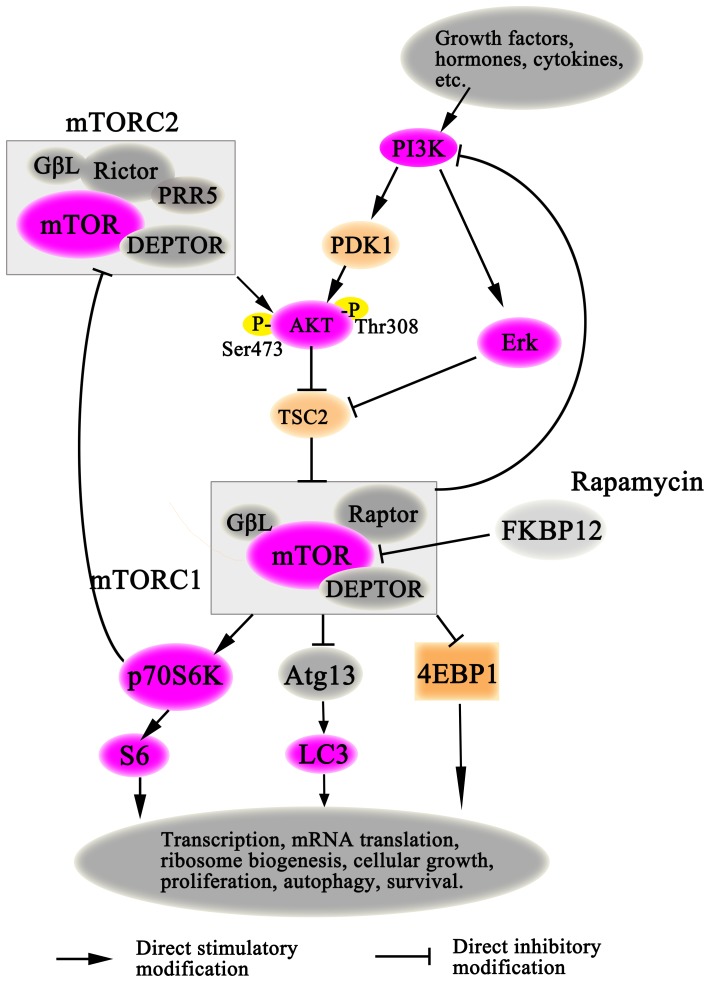
PI3K/AKT/mTOR signaling pathway. Rapamycin bound to FKBP12 mainly blocked the activity of mTORC1.

In order to better explain our research, we based on previous studies to draw a schematic diagram of AKT/mTOR signaling pathway in Figure 9 [9], [12]–[14]. To our knowledge, we for the first time demonstrated that the AKT/mTOR signaling pathway was significantly overactivated and mTORC1 rather than mTORC2 was inhibited by rapamycin in the early phase of cirrhotic portal hypertension in rats (Figure 9). We evaluated the mRNA expressions of mTOR, P70S6K and 4EBP1, but no significant differences were obtained from all of the four groups. These results suggestted that mTOR signaling molecules had no obvious change at the transcription level, and rapamycin showed no evident influence on their mRNA expressions in the early phase of cirrhotic portal hypertension. Afterwards, we consistently detected the expressions of p-AKT (Ser473/Thr308), p-mTOR, p-P70S6K and p-S6 relative to their total proteins in BDL rats, all of which were profoundly higher than those in SHAM rats, indicating the activation of AKT/mTOR signaling pathway. However, treatment with rapamycin significantly inhibited the expressions of p-P70S6K and p-S6 but not p-AKT (Ser473) to their total proteins in BDL-Ra rats. Since p-P70S6K and p-AKT (Ser473) are the direct downstream effectors of mTORC1 and mTORC2 respectively (Figure 9), it indirectly proved that rapamycin blocked mTORC1 but not mTORC2, which was consistent with the researches of Sarbassov and Feldman [2], [17]. Whereas, investigations in vitro suggested rapamycin treatment for more than 24 h might also inhibit mTORC2 assembly to suppress AKT/PKB (Ser473) phosphorylation in certain cell lines [38]. In our study, however, the relative expressions of p-mTOR and p-AKT (Ser473) were still much higher in BDL-Ra livers than those in SHAM. These results suggested treatment with rapamycin (2 mg/kg/day) for two weeks did not block mTORC2 assembly, nor did it reduce the levels of mTORC2 below those needed to maintain AKT/PKB signaling in this pathophysiologic progress of cirrhotic portal hypertension.

While mTOR activation is a downstream effector of AKT, mTORC1 reciprocally regulates the growth-factor responsiveness of PI3K and AKT through feedback inhibition (Figure 9) [39], [40]. As is known, AKT/PKB activation requires the phosphorylation of Thr308 and Ser473 [2]. Our data showed that the relative expressions of p-AKT (Thr308) and p-AKT (Ser473) were still much higher in BDL-Ra rats than those in SHAM group. Thus it was possible that inhibition of mTOR and P70S6K by rapamycin partly induced AKT Ser473 and Thr308 phosphorylation in BDL-Ra rats by feedback (Figure 9).

On the other hand, the MAPK/ERK signaling cascade, which belongs to the mitogen activated protein kinases (MAPKs) family, is another major player in the mitogenic and antiapoptotic response in many cells [16], [20], [41]. Existing researches show that MAPK/ERK and AKT/mTOR signaling pathways are colsely related in cholestatic liver injury [20], [41]. They are two parallel regulatory pathways to control cell survival, differentiation, proliferation, metabolism, and motility in response to extracellular cues [42]. However there also exists an important cross-talk, in which ERK modulated mTOR signaling through phosphorylation and inactivation of tuberous sclerosis complex 2 (TSC2) (Figure 9) [43].

Our study clearly showed that both AKT/mTOR (Figure 7) and ERK1/2 (Figure 8) signaling cascade were profoundly overactivated in livers of early cholestatic fibrosis, indicating both of them were closely involved in the formation of this disease. However, we found that treatment with rapamycin had no obvious influence on the expression of p-ERK1/2 to ERK1/2, but distinctly inhibited the expressions of p-P70S6K and p-S6 to their total proteins. The expression of p-mTOR to mTOR in BDL-Ra rats was still significantly higher than that in SHAM animals (Figure 9). Therefore, it was also possible that the high expression of p-ERK1/2 to ERK1/2 might stimulate the activation of mTOR to partly antagonize the inhibitory effect of rapamycin in this disease. Collectively, these data indicated that MAPK/ERK and AKT/mTOR signaling pathways might participate in the disease in a collaborative manner. A further investigation with a single or joint specific inhibition of the two pathways is needed to explore the exact mechanisms.

Another finding of the present study was that rapamycin markedly decreased the body weight in both SHAM-Ra and BDL-Ra rats compared with their controls. mTOR pathway mainly regulates protein synthesis, transcription, cell growth, cell proliferation, cell motility, and cell survival in almost all the cells [9], [12]–[14]. Thus mTOR blockade by rapamycin may slow down the normal growth of rats. Other study also described weight loss of rats caused by rapamycin at a low dosage 0.2 mg/kg/d [44]. Therefore, it was not surprising to find the similar phenomenon in our study. Despite the loss in body weight, all the rats in SHAM-Ra group seemed healthy. Moreover, rapamycin is a FDA–approved immunosuppressive drug to inhibit transplant rejection in clinical application. Therefore, rapamycin should be a safe and stable drug in this study.

In conclusion, this study demonstrated that the activation of AKT/mTOR signaling pathway was clearly involved in the pathophysiological onset of early cirrhotic portal hypertension in rats. Early treatment with rapamycin did effectively ameliorate intrahepatic inflammation and fibrosis, improve liver function, decrease splenomegaly and portal pressure, as well as reduce the expressions of p-AKT T308, p-P70S6K and p-S6 relative to their total proteins. These evidences indicated the essential role of AKT/mTOR signaling pathway in the early pathophysiologic progress. The supression of p-P70S6K and p-S6 but not p-AKT S473 to their total proteins by rapamycin indirectly confirmed that it mainly inhibited mTORC1 rather than mTORC2 in the early phase of cirrhotic portal hypertension.
